# Pharmacogenetics and its impact on pharmacological management of severe attention deficit hyperactivity disorder

**DOI:** 10.1192/j.eurpsy.2025.1169

**Published:** 2025-08-26

**Authors:** M. Okružnik Želalić, I. Stefanović, I. Žunić Išasegi, L. Šimičević, L. Ganoci, M. Živković, I. Begovac

**Affiliations:** 1 Department of Child and Adolescent Psychiatry; 2 Department of Laboratory Diagnostics, University Hospital Center Zagreb; 3 School of Medicine, University of Zagreb; 4 Department of Psychiatry, University Hospital Center Zagreb, Zagreb, Croatia

## Abstract

**Introduction:**

Attention Deficit Hyperactivity Disorder (ADHD) varies in presentation and associated comorbidity conditions. Diagnosis and treatment is often challenging, highlighting the need for individualized approaches in managing ADHD. Optimal therapy includes a combination of different methods such as psychological interventions and pharmacotherapy. Pharmacogenetics allows for a more personalized and effective treatment plan, which can reduce empirical prescribing of medication, meaning less side effects, faster treatment response and achieving remission. Altogether, this leads to improved compliance and outcomes.

**Objectives:**

In our opinion this is an interesting case study which explores the challenging presentation, medical management, and treatment of a patient with ADHD and its comorbidity.

**Methods:**

We present a male subject, age 14, who met DSM- V criteria for ADHD at the age of 8. The treatment effects of conventional approaches with psychosocial interventions and individual psychotherapy as well as child and parental psychoeducation had not proven sufficient, so pharmacotherapy was added to the treatment strategy. He was initially introduced to methylphenidate therapy and developed side effects in the form of depressive symptoms and motor tics. Regression of side effects occurred when the drug was discontinued. Impulsive and aggressive behaviors became severe so antipsychotics were prescribed, which resulted in improvement of behaviour. Attention and concentration disturbances remained, however. During this period, the subject experienced a growth spurt, gained in body weight and his laboratory findings showed high liver enzymes. We conducted a multidisciplinary approach that included a complete examination by a geneticist, an endocrinologist, and a cardiologist. EEG and psychological testing were performed. Due to a lack of progress in socio-emotional functioning, genotyping analyses of CYP2D6, CYP1A2, CYP2C9, CYP2C19, CYP3A4, CYP3A5, ABCB1, ABCG2, 5-HTTLPR, DAT1 VNTR was performed.

**Results:**

The pharmacogenetic findings suggested a higher activity of the CYP2D6 enzyme than normal. Significantly reduced and weak transport function of protein ABCB1 was observed. Atomoxetine is not a substrate for ABCB1, so the introduction of atomoxetine is planned after the stabilization of liver enzymes.

**Conclusions:**

Various treatment strategies can help ameliorate ADHD symptoms. Finding an effective medication and dosage for a given child with ADHD can be a complex process. Although pharmacogenetic testing is not a standard procedure in child and adolescent psychiatry, it can have an impact on the management of treatment-resistant symptoms and medication-related side effects. The potential for pharmacogenetics to enhance treatment precision remains a promising area for future research in psychiatry.

**Disclosure of Interest:**

None Declared

